# Some Facts Showing the Relationship of the Dental Interarticulation, with More or Less Obscure Pains about the Mouth and Jaws

**Published:** 1893-10

**Authors:** L. Van Orden

**Affiliations:** San Francisco, Cal.


### SOME FACTS SHOWING THE RELATIONSHIP OF THE DENTAL INTERARTICULATION, WITH MORE OR LESS OBSCURE PAINS ABOUT THE MOUTH AND JAWS.[1]

BY L. VAN ORDEN, M.D., SAN FRANCISCO, CAL.

In April, 1889, Mr. W. G. L. presented himself with acute pericementitis of the left superior cuspid, which possessed dense structure, with a fair-sized gold filling upon its distal aspect. At the time a pulp-lesion was not presumed to exist; but as it was observed that some irregularity was present, and that the occlusion was not normal, an engine-stone was used to relieve the tooth of undue work and the patient of an amount of pain that almost seemed to demand recourse to extraction. No abscess formed, and the recovery was so rapid as to fix the case in the writer's mind without the aid of any memorandum. An examination of the case after four years shows no recurrence of any trouble, but discoloration seems to point to death of the pulp.

On Christmas-day, 1891, Mr. C. B., aged about forty-five years, of bilio-nervous temperament, called upon the writer at his residence, with an acute abscess pointing upon the lingual aspect of the socket of the right inferior third molar, well below the gingival margin. Temporary relief was afforded by evacuation, and at several subsequent sittings, type-writer's carbon paper was made use of and the occlusion of the tooth with the superior second and third molars rendered comfortable. By reference to these models, which are those used in illustration of Dr. Dean's paper, published in 1892 in the *Dental Cosmos*, as a reply to the paper by Dr. Davenport, of Paris, on the dental interarticulation, it will be observed that the right lower first molar and second bicuspid had suffered extraction some years previously. While Dr. Dean pointed out several ill effects that had seemed to follow these extractions, including no-

---

[1] Read before the World's Columbian Dental Congress, Chicago, August, 1893.

ticeable cupping of the right cuspids and bicuspids and pyorrhœa alveolaris of the right superior central and lateral incisors involving a loss of their proximal process to the depth of about one-half inch, it seemed probable that the forward migration of the right inferior second and third molars had given rise to slight but repeated shocks or insults to the latter tooth. The *cup-and-saucer* form of the interarticulation of the right third molars may here be adverted to. The distal edge of the saucer has seemed, in a number of cases, to be especially concerned in producing the slight shocks which resulted in such severe inflammation. Though this abscess was the third one that had formed, no other trouble was complained of by the patient until some eighteen months later, when an abscess having formed on the buccal aspect, the tooth was extracted.

A Mrs. B. had been suffering with a slight dull and quiet continuous pain in the region of the left superior third molar. Several dentists had been consulted, and ascribed such causes as a "slight cavity, too small to fill," and "a dimple" (exostosis?). The tooth was slightly sensitive to percussion; the first lower molar was missing, and the cup-and-saucer condition of interarticulation was noticeable, and profiting by the previous case, the distal edge of the saucer was reduced and immediate relief was acknowledged.

A Mrs. D., aged about twenty-three years, had an exposure of the pulp of the left inferior first molar, which was disclosed upon the removal of a large and defective amalgam filling, pain being present. The pulp was devitalized and the canals medicated and filled. The patient returned complaining of discomfort. The left superior first molar had been extracted. As the left superior and inferior molars were sensitive to percussion, their interocclusion was eased and the trouble ceased. It seemed at the time that part of the discomfort was caused by lateral pressure of the left inferior second molar upon the inferior first molar, thus keeping up an irritability of the pericementum of the latter tooth.

Mr. F. V., aged twenty-four years, had for many months suffered discomfort in mastication with the left superior second molar; an itching sensation in the tooth was also complained of. The patient acknowledged a great sense of relief when the force and direction of the impact had been modified, and has had no return of the symptoms in more than a year.

The foregoing cases have been significantly associated with the loss of one or more teeth through extraction. The case of Mr. L. C., aged thirty-five years, is associated with a comparatively normal denture. After the large bucco-morsal cavity of the left inferior second molar had been successfully filled, tenderness was noticed in the first molars of the same side. The discomfort seemed to be due to the prominence of the cusps of these teeth, and comfort was restored by the use of test-paper and the corundum-stone.

Reference having been made to the "cup-and-saucer" form of inter-occlusion of the third molars, the writer has ventured to refer the same to a pivoting action of the masticatory apparatus, sometimes more pronounced on one side than on both. A Scotch gentleman, about thirty years of age, called in 1881. The only defects to be remedied were two small cavities on the buccal and morsal aspects of the left inferior third molar. Gold fillings were inserted, which, in time, were dislodged; and both amalgam and cement fillings were resorted to, from time to time, without success, until now we have the deep, smooth saucer-shaped surface in the lower tooth with its converse in the upper tooth. (The concavity will sometimes be found to exist in the superior tooth, and the convexity in the lower one.) That there is and perhaps always has been disproportionate muscular action, in this case, on one side, is somewhat supported by the fact that, in 1888, the lingual portion of the crown (non-carious) of the left inferior second molar was broken off by biting upon a small piece of bone in the food. The pulp, being exposed by the accident, was devitalized by arsenic, the canals were filled with a medicated paste, and the crown restored with amalgam. In July, 1893,—five years later,—this tooth was found to be again fractured longitudinally through its buccal half, and was extracted as being beyond further aid.

The main benefit that the writer has derived from the observation of these and other cases has been the conviction that the extraction of one or more teeth, especially of the inferior first molars, is liable, sooner or later, to lead to discomfort in some of the remaining teeth, and that such discomfort may, with patience, be located and relieved by the use of an engine-stone.

